# Hyaluronic Acid in the Intestinal Tract: Influence of Structure, Rheology, and Mucoadhesion on the Intestinal Uptake in Rats

**DOI:** 10.3390/biom10101422

**Published:** 2020-10-08

**Authors:** Alexandro Barbosa de Souza, Marco Vinícius Chaud, Thais Francine Alves, Juliana Ferreira de Souza, Maria Helena Andrade Santana

**Affiliations:** 1 Department of Materials and Bioprocesses Engineering, School of Chemical Engineering, University of Campinas, P.O. Box 6066, Campinas 13083 852, SP, Brazil; souzaozzy@yahoo.com.br; 2 Laboratory of Biomaterials and Nanotechnology, University of Sorocaba, Sorocaba 18300 000, SP, Brazil; chaudmarco@gmail.com (M.V.C.); thaisfrancine1@hotmail.com (T.F.A.); julianafsz@yahoo.com.br (J.F.d.S.)

**Keywords:** hyaluronic acid, nanoparticles, tack adhesion, intestinal permeability

## Abstract

Oral hyaluronic acid (HA) is a ubiquitous biopolymer that has gained attention as a treatment for local or systemic diseases. Here, we prepared and characterized structures of free HA (f-HA) with a high (>10^5^ Da), intermediate (≤10^5^ Da), and low (≤10^4^ Da) average molar mass (MM); nanoparticles crosslinked with adipic dihydrazide (n-HA); and mixed formulations (mixed-HA) containing f-HA and n-HA. MM distribution determined the structure, hydrodynamic diameter, and zeta potential of the f-HAs. Crosslinking changed the physicochemical properties in n-HA. In vitro tack adhesion assays, using mucin tablets or a viable rat intestinal mucosa, showed better mucoadhesion with f-HA (intermediate MM) and mixed-HA (25% n-HA), especially in the jejunum segment. High MM f-HA presented negligible mucoadhesion. n-HA showed the deepest diffusion into the porous of the membranes. In vivo results showed that, except for high MM f-HA, there is an inverse relationship between rheological changes in the intestinal membrane macerates resulting from mucoadhesion and the effective intestinal permeability that led to blood clearance of the structures. We conclude that the n-HA formulations are promising for targeting other tissues, while formulations of f-HA (intermediate MM) and mixed-HA are better for treating dysbiosis.

## 1. Introduction

Hyaluronic acid (HA) is a glycosaminoglycan ubiquitous in the human body [1]. HA performs biological functions at various concentrations, molar masses (MM), and size distributions of its chains structured in different spatial architectures [2,3,4,5]. Then, at the intestinal mucosa, intermediate and high MM (≥10^5^ Da) present antioxidant and anti-microbial properties [6,7]. While the integrity of polymeric HA structures regulates tissue homeostasis, the decrease of its endogenous production or cleavage of its chains is linked with various diseases related mainly to age. Nowadays, the HA from fermentation (bio-HA) is widely used to restore the endogenous HA properties that have been lost [8,9].

The TSG-6 (Tumour Stimulate Gene-6) is a 35-kDa HA-binding protein that is usually expressed in response to proinflammatory stimuli is an important protector of tissue structure during inflammation. The effect of multifunctional protein TSG-6 in various models of inflammation shows an alteration the structure of HA via its direct crosslinking of HA chains, enhance HA-receptor interactions and contributes to the anti-inflammatory effects. This anti-inflammatory process by an allosteric mechanism preventing simultaneous binding of HA and inhibition of interleukin-1 (IL-1), interleukin-6 and tumour necrosis factor (TNFα), and competing interactions of HA for their distinct binding sites on link_TSG6, both process are more effective with HA with high MM [10,11].

Oral administration of exogenous HA has gained attention from researchers as a supplementary or complementary therapy to prevent or treat diseases. Over the last four decades, it has been postulated that a low and incomplete fraction dose of ingested HA is absorbed as a high molar mass (MM ≥ 10^5^ Da) [8,12,13]. However, the reported studies were generally qualitative, based on fluorescent methods for the identification of HA in tissues. Additionally, mucoadhesion can be affected by the properties of HA structure related to hydration, structural stability, and surface charge [14,15]. A study using animal model demonstrated that after intestinal damage that HA homeostasis in myenteric neuron influence the intestinal transit, improve the disorder of myenteric plexus during intestinal inflammation and hinders the development of dysmotility [16,17]. However, studies focusing on the influences of HA structuring on its intestinal adhesion and uptake are scarce in the literature. Therefore, the knowledge about the intestinal uptake of HA needs to be advanced in terms of kinetics and HA structuring.

In this study, the influence of HA structuring on its penetration into type III mucin of a porcine stomach and rat intestinal mucosa was studied using free HA (f-HA), nanoparticles crosslinked with adipic dihydrazide (n-HA), and mixed HA (mix-HA) formulations. The selected formulations from an in vitro study (f-HA average molar mass 10^5^ Da, n-HA with 475.3 ± 75.8 mean diameter as well as a mix-HA containing 25 wt % nanoparticles dispersed in the f-HA) were investigated using the improved single-pass intestinal permeability (SPIP) method [18,19,20], which considers the entire small intestine of rats to assess the effects of HA structuring on its intestinal permeability. The results highlighted the importance of HA structuring on mucoadhesion, facilitating the selection of promising formulations with improved penetration properties for HA oral administration and investigation in pre-clinical assays.

## 2. Materials and Methods 

### 2.1. Polymers and Chemicals

Hyaluronic acid sodium salt at 1% (*w/v*) was purchased from Mapric Pharmaceutical Products Ltd., (São Paulo, Brazil) (I-MM) and Euflexxa (São Paulo, Brazil) (H-MM). The HA from Mapric was used in the synthesis of HA nanoparticles. Water-soluble adipic acid dihydrazide (ADH), N, and *N*-dimethyl aminopropyl carbodiimide (EDC) were used for nanoparticle crosslinking and were purchased from Sigma (St. Louis, MO, USA). Ethanol and all other analytical grade chemicals were acquired from Merck (Darmstadt, Germany). Type III mucin (Sigma-Aldrich Ltd., São Paulo, Brazil) in partially purified powder from porcine stomach was used in the mucoadhesion assays. 

### 2.2. Pre-Treatments and Characterization of Free HAs

The HA Euflexxa, distributed by the manufacturer into sterile syringes for medical use, was used without any pre-treatment. HA Mapric sold for cosmetic use was precipitated with ethanol for purification before being used. 

Precipitation was carried out with ethanol at pH 7.0 in the presence of NaCl (2 M), according to the protocol described by Cavalcanti et al. [21]. The precipitate was separated by centrifugation at 1318× *g* for 20 min and suspended in NaCl (0.15 M) for stabilization. 

The precipitate was later hydrolyzed to provide the low average MM with the same purity grade. HA hydrolysis was carried out resuspending the precipitated HA in a phosphate buffer (0.1 M) at pH 12. The obtained solution (5 g/L) was left at 60 °C for 24 h in a reciprocal shaker bath with 500 rpm stirring speed (Lab-Line Instruments Inc., Melrose Park, IL, USA). After that, the hydrolyzed HA was freeze-dried for storage. The free HAs were characterized in terms of concentration, purity, average MM, and distribution. 

#### 2.2.1. Purity and Concentration

The purity relative to proteins was determined from HA and protein concentrations according to Equation (1):

(1) $$P=\frac{C_{\mathrm{HA}}}{\left(C_{\mathrm{HA}}+C_{\mathrm{SP}}\right)}\times 100$$

where $C_{\mathrm{HA}}$ and $C_{\mathrm{SP}}$ are the concentrations of the HA and soluble protein, respectively.

The HA concentration was quantified by the CTAB method [22]. Briefly, HA solutions and CTAB were mixed at 0.5/1 (*v*/*v*). The mixture was maintained for 10 min at room temperature, after which the optical density was measured at 400 nm wavelength. The spectrophotometer was calibrated using a control solution prepared from NaCl (0.15 M) and CTAB at the same ratio. Analytical grade sodium hyaluronate (HylumedTM) from Genzyme Corporation (Cambridge, MA, USA) was used as a standard for the calibration curve. 

The protein content was quantified by bicinchoninic acid (BCA) using an assay kit from Sigma-Aldrich, St. Louis, MO, USA). Briefly, the precipitate and BCA reagent were mixed at a 1/20 (*v/v*) ratio and incubated in a water bath at 37 °C for 30 min. After reacting, the optical density was measured at a wavelength of 562 nm. The protein concentration was determined from a standard curve previously constructed with bovine serum albumin (BSA).

#### 2.2.2. Average Molar Mass and Distribution

The average molar mass (MM) of the f-HAs was determined by size exclusion chromatography using a gel filtration column (Polysep-GFC-P6000, 7.8 mm × 300 mm; Phenomenex, Torrance, CA, USA) coupled to a Shimadzu RID-6A refractive index detector (Shimadzu Corporation, Kyoto, Japan). Briefly, 20 μL of (0.01 g/L) free HA at pH 7.4 was injected using NaNO_3_ (0.1 M) as a mobile phase at 1.0 mL min^−1^ and a temperature of 25 °C. HA analytical standards (Hyalose, Oklahoma, OK, USA) with MM ranging from 50 to 1000 kDa were correlated with retention time using Equation (2):

log MM = 10.95 − (0.62 × retention time).(2)

The MM distribution was calculated considering Equation (2) and the area under the curves of the chromatographic peaks relative to 10^6^, 10^5,^ and 10^4^ Da. The free HAs were classified according to the average MM as high MM (H-MM)—richer in the 10^6^ and 10^5^ Da fractions; intermediate MM (I-MM), richer in the 10^5^ Da; and low MM (L-MM), which was richer in 10^4^ Da fractions.

### 2.3. Preparation of HA Crosslinked Nanoparticles

Crosslinked HA nanoparticles were prepared from free I-MM according to the protocol described by Hu et al. [23], with modifications from Bicudo et al. [24]. Nanoparticles were formed by HA precipitation due to its local dehydration. Briefly, the process was carried out in a jacket glass reactor (400 mL) under a controlled temperature of 21 °C and gentle mechanical stirring. Ethanol (Merck, Darmstadt, Germany) was incorporated at a flow rate of 7 mL/min into a 0.1% (*w*/*v*) solution of sodium hyaluronate with an average MM 10^5^ Da (Mapric Pharmaceutical Products Ltd.). Initially, an ethanol volume of approximately 100 mL was added and the reacting system was maintained under stirring for 2 h. Next, EDCI (40 mg/mL) and ADH (20 mg/mL), both in aqueous solutions, were added to the reactor for crosslinking during 24 h stirring. The reaction was concluded by the addition of more ethanol volume (100 mL approx.) and a further 20 h stirring. Nanoparticles were recovered from the dispersion by ultrafiltration in an Ultracel^®^ cell, containing a 10 kDa ultrafiltration disc and a 44.5 mm diameter filter (EMD Millipore Co., Billerica, MA, USA), operated under a 0.5 psi inlet nitrogen pressure. The yield (Y) of the nanoparticle preparation was evaluated using Equation (3):

(3) $$Y\;\left(\%\right)=\frac{\left(T_{m}-F_{m}\right)}{T_{m}}\times 100$$

where $T_{m}$ is the total mass, calculated by the sum of the masses in the filtrate (*Fm*) and retentate ($T_{m}-F_{m}$), which contained the nanoparticles.

### 2.4. Preparation and Characterization of HA Colloidal Dispersions

The colloidal dispersions of the free HA (f-HA) were prepared directly from the medical-grade syringes, Euflexxa HA (H-MM), and post-ethanol precipitation of the dermocosmetic grade Mapric HA (I-MM). Additional hydrolysis of I-MM was performed to obtain L-MM. The preparations were obtained in dilute 0.01 g/L and a semi-diluted state in phosphate buffer at pH 7.4 under different concentrations. The nanoparticle dispersions (n-HA) were prepared under the same conditions. The HA-mixed dispersions (mixed-HA) were prepared from the freeze-dried powder of I-MM and mixed with 25% or 50% (wt.) nanoparticles to obtain 10 g/L phosphate-buffered pH 7.4 dispersions. The mixtures were stirred overnight at 37 °C to ensure the reproducibility of the preparations. The colloidal dispersions were characterized by the apparent viscosity and oscillatory rheology.

#### Rheological Characterization

The rheological behavior of the prepared dispersions was characterized using an Anton Paar MCR-102 Modular Compact Rheometer. The assays were conducted using a cone-plate geometry (CP50-1) with a 50 mm diameter, a cone angle of 0.9815°, and a truncation of 0.97 µm. The measurements were performed at 37 °C. Steady-state shear and oscillatory measurements were performed at shear rates from 0.01 to 1000 s^−1^ and an angular frequency from 0.1 to 600 rad s^−1^, respectively. The apparent viscosity was determined at 25 °C using a Vibro-SV-10 viscosimeter (Tokyo, Japan).

### 2.5. Characterization of HA Structures

Physicochemical characterization of the HA colloidal structures present in the dispersions was carried out based on the mean hydrodynamic diameter, size distribution, and zeta potential. Biological characterization was performed by in vitro mucoadhesion assays and in vivo intestinal perfusion. 

#### Mean Hydrodynamic Diameter, Size Distribution, and Zeta Potential

The mean hydrodynamic diameter, size distribution, and polydispersity index (PDI) were determined by dynamic light scattering (DLS) and the data were analyzed by photon correlation spectroscopy (PCS). Measurements were performed with a 4 mW HeNe Laser (633 nm) in an Autosizer 4700, Zetasizer Nano Series (Malvern, Malvern, UK), at a fixed angle of 173° and a temperature of 25 °C.

The mean hydrodynamic diameter was expressed as the Z-average:

(4) $$Z\text{-}\mathrm{average}=\sum \frac{n_{i}d_{i}^{3}}{n_{i}d_{i}^{2}}$$

where n is the number of particles with a given diameter, *d*.

Size distributions were analyzed in terms of the spectra of the intensity of scattering, proportional to the diameter, *d*, to the power of six (I-distribution α *d*^6^) for the various size classes and the particle distribution (N-distribution α *d*), which is proportional to the diameter, to obtain the predominant sizes [25].

The zeta potential was determined by measurements of the electrophoretic mobility at a set potential, using a 4 mW HeNe Laser (633 nm) in an Autosizer 4700, Zetasizer Nano Series (Malvern, Malvern, UK).

### 2.6. Mucoadhesion

Mucoadhesion assays were carried out with two models of biological substrate: Type III mucin of a porcine stomach and fresh tissue from rat intestinal mucosa.

Type III mucin of the porcine stomach is a partially purified powder, containing 0.5–1.5% of sialic acid bound to a glycoprotein polymer. Mucin tablets were manufactured by machine compression (LM-D-8, Lemaq, São Paulo, Brazil). Tablets in the dry form with a plane and smooth surface (120 mg, 8 mm diameter, and 2.2 ± 0.12 mm thickness) were obtained according to the principles described in the Brazilian Pharmacopoeia reference standards. The friability and hardness of the mucin tablets were 2.9% ± 0.4% and 37.5 ± 3.8 N, respectively. Loss of mass by friability and hardness were 0.3% ± 0.08% and 8 ± 0.11 Kg, respectively.

The intestinal mucosa of rats was extracted from male Wistar rat *Mus norvegicus albinis* sourced from a local supplier (Anilab, Paulínia, Brazil). The study with animals of this work was approved by the Animal Ethics Committee of the University of Sorocaba, São Paulo, Brazil (application number 091/2016).

Preparation of the animal substrate followed the following protocol: Adult male rats of the same age and weighing 260–280 g fasted for 10 h and were provided with water ad libitum. The operative procedure began with intraperitoneal anesthesia using thiopental sodium (0.05 mg/100 g body weight). The small intestine was exposed after a longitudinal scission of the abdomen. A non-traumatic hemostatic tweezer was used to facilitate the scission and occlusion of the duodenum proximal (0.5 cm below the pylorus) and duodenum distal (8.5 cm below the pylorus). Then, the mesentery over 8 cm length duodenum was carefully removed. Fresh tissue sections (duodenum, jejunum, and ileum) were cut off in approximately 10 cm^2^ pieces. Each segment was opened lengthwise, everted with the aid of a flexible rod (2.5 mm in diameter) with an end covered by fine silk fabric and gently washed with 0.9% NaCl solution at 37 °C. All mucosa was visualized for integrity and viability.

The mucoadhesive properties of the HA formulations were investigated using a TA-XTplus texture analyzer (Stable Micro Systems, Surrey, UK) [26]. Mucin tablets were preconditioned by pre-swelling with purified water at 37 °C for 15 min [27], and the fresh intestinal mucosa was used after washing with 0.9% NaCl solution at 37 °C. The mucin tablets and tissue sections were horizontally attached to the lower end of the analytical P/10 probe using double-sided adhesive tape and suture thread.

Selected HA dispersions with the f-HA, n-HA, and mixed-HA structures were placed into a constant volume compartment and conditioned in a water bath at 37 °C. For intimate contact with the substrates, the analytical probe descended onto the surface of each dispersion, at a constant speed of 0.2 mm/s, along a penetration length 10 mm, and a downward tensile force (F) of 0.49 N was applied for 3 min. The biological substrate returned vertically to the surface at a constant speed of 5.0 mm/s. After every cycle, the HA dispersions and the biological substrate were replaced. The force versus distance profiles provided the peak force, the maximum force of detachment (F_adh_), and the work of adhesion (W_adh_) or adhesion energy as the integral of the resulting force–distance profile.

Statistical analysis of the responses of adhesion was carried out using one-way analysis of variance (ANOVA) with post-hoc Tukey. A significance level of *p* < 0.05 was used. Values were expressed as the mean ± standard deviation (SD) of at least three replicates in mucin tablets and six replicates in fresh tissue sections.

### 2.7. Intestinal Perfusion

The in vivo intestinal perfusion assays were performed on rats. Anesthesia, surgical, and perfusion procedures were justified in detail and were approved by the Animal Ethics Committee of the University of Sorocaba, São Paulo, Brazil (application number 091/2016), following the guidelines described in the Brazilian national laws governing the use of animals in research. *Mus norvegicus albinis* rats (male, 260–280 g in weight, age 8–10 weeks) were kept in a 12 h light/dark cycle at 25 °C and 50% relative humidity. They came from the same local supplier (Anilab, Paulínia, Brazil) and were kept under the same diet and housing conditions.

Twelve healthy rats were divided into four groups randomly, with three rats in each group. The four groups were as follows: (1) Normal saline (NS) control group; (2) free HA group; (3) mixed HA group; and (4) nanoparticulate HA group. 

Three HA dispersions were used in the perfusion experiments: (I) (f-HA) I-MM HA 10 g/L prepared from a 1% (*w/v*) sodium hyaluronate solution; (II) (n-HA) 2.5 g/L; (III) mixed-HA 25 wt.% n-HA to a final concentration of 10 g/L; NaCl 0.9 wt.% was used as a control in the perfusion assays.

### 2.8. HA Concentration Analysis

#### 2.8.1. Analysis in the Perfusate

The analysis of the HA concentration in the perfusate was carried out according to the protocol described in Section 2.2.1.

#### 2.8.2. Analysis in the Permeated

The blood of rats was drawn from the brachiocephalic vein at a variety of time points and centrifuged after serum separation. Afterwards, a HA ELISA-like assay was used to quantify the amount of HA from the serum. The detection range varied from 1.56–100 ng/mL and presented sensitivity <0.94 ng/mL.

For the analysis, samples of serum were initially allowed to clot for 2 h at room temperature, then centrifuged for 20 min at 1000× *g*. The serum pool was collected and immediately frozen and stored at −20 °C before analysis. Samples of serum and standards were incubated with a biotinylated detection antibody in a 96-well microplate that had a fixed amount of HA immobilized on the bottom of each well for 45 min at 37 °C. During the reaction, HA in the samples or the standard competed with a fixed amount of HA on the solid phase supporter for sites on the biotinylated detection antibody specific to HA. Antibodies and the sample/standard were washed from the plate, and streptavidin conjugated to horseradish peroxidase (HRP) was added to each microplate well and incubated for 30 min at 37 °C. After the washing steps, the HRP substrate tetramethylbenzidine (TMB) was added to each well and incubated for 15 min at 37 °C. The enzyme–substrate reaction was terminated by the addition of a sulphuric acid solution, and the absorbance of the sample was measured at 450 nm wavelength. A standard curve relating the absorbance and HA concentration was constructed and adjusted to a linear regression. A blank containing only the diluent was used to correct for background absorbance.

#### 2.8.3. HA Molar Mass Distribution and Particle Size Distribution

The molar mass distribution of HA in the ingoing and outgoing perfusate was determined as described in Section 2.2.2.

#### 2.8.4. Rheological Characterization

Rheological measurements of macerated small intestine were carried out at 37 °C as described in Section “Rheological Characterization”. 

#### 2.8.5. Improved In Situ Single-Pass Intestinal Permeability

The improved in situ perfusion study was performed using an established method [28], with minor modifications. The rats were fasted overnight (10 h) and provided water ad libitum. Then, rats were anesthetized with an intraperitoneal injection of thiopental sodium (0.05 mg/100 g weight body), placed on a table, and maintained at 37 °C. The abdomen was opened by a 3−5 cm longitudinal incision along the midline, and the intestines were exposed. 

Proximal and distal ends of the small intestines were identified, incised, and cannulated using silicone tubing (O.D. 4 mm, I.D. 2 mm) for inlet and outlet perfusion, respectively. The entire length of the small intestine, ranging from 88 cm to 96 cm, was used. The intestine was carefully placed back into the peritoneal cavity, and the abdomen was covered with parafilm to prevent peritoneal dehydration. Cotton wool pads prevented heat losses leaving the inlet and outlet tubing accessible from the outside. This set up ensured the isolation of the small intestine, and HA aqueous dispersion can be introduced and sampled with the aid of the peristaltic pump and stopcock valves.

Inlet tubing was attached to the peristaltic pump (Petro Gas Ausrüstungen, Berlin, Germany). Saline solution (37 °C) was gently pumped through the inlet tubing until it filled the whole intestine to remove adherent and non-adherent particulates present inside the intestinal lumen. The procedure lasted 15 min until the outlet is clear. Then, at the starting point of each experiment, a 17.6–19.2 mL of 37 °C perfusate formulation was infused (5 mL/min) to fill the entire segment. The small intestine was fed under a steady-state regime with a flow rate varying with the used formulation depending on its viscosity. Free HA with high viscosity (349 mPa·s) was pumped at a flow rate of 0.5 mL/min. The other two formulations (mixed and nanoparticulate HA) of low viscosity (1.5 and 1.8 mPa·s, respectively) were pumped at a flow rate of 0.2 mL/min.

Blood was collected following a pre-established schedule of 6, 30, 60, 90, and 120 min in continuous perfusion. The samples of blood were manipulated according to the description in Section 2.8.2.

Weight and volume measurements quantified the net water flux (NWF). NWF is the ratio of the volume of perfusion solution in the outgoing perfusate to the volume of perfusion solution in the ingoing perfusate. Steady-state levels of HA in the perfusate volumes were reached after 60–90 min.

At the end of the experiment, the intestine was emptied with air pressure, and the rats were euthanized by a single lethal intracardiac dose of anesthetic (thiopental sodium). The length of the intestinal segment was measured and macerated using a digital disperser Ultraturrax (T-25—IKA Works GmbH & Co. KG, Staufen, Germany) for further rheological measurements. 

The effective permeability coefficient (P_eff_) of HA structures was calculated using the parallel-tube model [29] from the steady-state concentrations in the perfusate collected, after correcting for NWF.

Permeability values (mm/min) were calculated by Equation (5):

(5) Peff=Qin×⎣−ln CoutCinA⎦

where $Q_{in}$ is the flow rate; $C_{in}$ and $C_{out}$ are the inlet and outlet concentrations of HA, respectively; A=2πrL is the area of the exposed intestinal segment described as a smooth cylinder with a radius r (set to 0.2 cm) [30] and length L, measured after completion of the experiment.

All animal experiments were replicated with n = 3. The data are expressed as mean ± standard deviation (SD). Student’s *t*-tests were performed for experimental groups, and a value of *p* < 0.05 was considered significant.

## 3. Results

### 3.1. Structural Properties

The hydrophobic character of some parts of the polymer induces spontaneous aggregation in an aqueous medium which, added to the physical crosslinks of the entanglement chains, results in soft colloids with structural properties driving their functionality [31,32]. In the intestinal tract, the structural properties influence membrane interactions and uptake. The crosslinked HA nanoparticles (n-HA) have lower hydrophilicity and softness. The mixed system of free HA and crosslinked nanoparticles (mixed-HA) was considered for intermediate properties. 

Table 1 shows the physicochemical characterization of the structures in terms of zeta potential (ZP), hydrodynamic diameter (Z-average), and polydispersity index (PDI), as well as the percentages of distribution in the classes of 10^6^, 10^5^ and 10^4^ Da for H-MM, I-MM, and L-MM. Size spectra were recorded in terms of intensity (I) distribution that highlight all sizes present in the sample due to the signal amplification to diameter raised to the sixth power (I α d^6^) and the (N) distribution (N *α d*), where light scattering corresponds to the predominant network size (Supplementary Figures S1–S3).

### 3.2. Rheological Behavior

Rheological behavior plays an important role in the adhesion properties of the structures. For the required mucoadhesion at the intestinal membrane, the surface interactions of polymer–intestinal mucosa depend on the viscoelasticity of materials. The solid-like and liquid-like contributions perform together with the deformation on the surface and the energy dissipation, both of which are essential for adhesion. Therefore, the characterization of rheological behavior can predict adhesion properties [33].

**Figure 1:**
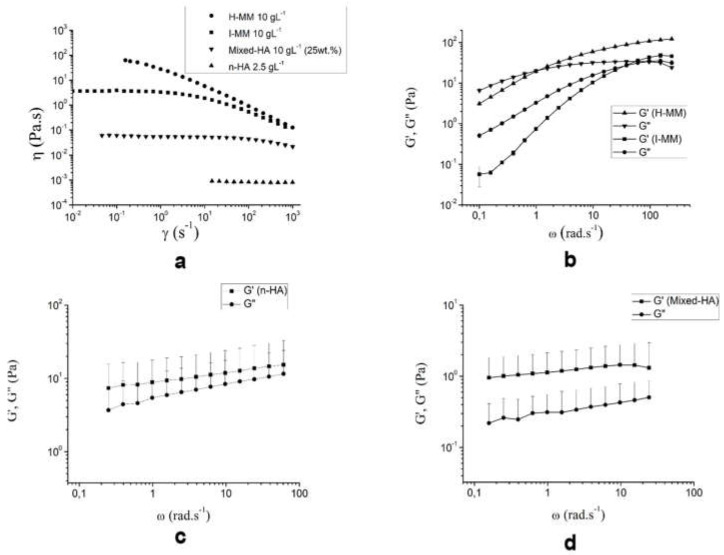
Rheological behavior of the free hyaluronic acids (f-HAs) with high (H-MM) (**a**) and intermediate MM (I-MM) molar mass (**b**), crosslinked HA nanoparticles (n-HA) (**c**) and mixed formulations (nanoparticles + free HA) (mixed-HA) (**d**). All formulations were buffered in pH 7.4 aqueous dispersions.

Linear oscillatory rheology provides the most sensitive measure of the critical gel point—the point when the material changes from a viscoelastic liquid to a viscoelastic solid. The gels are capable of storing and dissipating energy. Figure 1 describes the rheological behavior of the studied structures.

The curves of viscosity vs. shear rate shows the pseudoplastic nature and shear thinning behavior (decreasing viscosity) of the free HAs, while lower and constant viscosities were observed for n-HA and mixed-HA (Figure 1a). Figure 1b shows the evolution of the viscous or dissipative (G″) and elastic or energy storage (G′) modules as a function of frequency for the H-MM and I-MM f-HAs. Transitions (cross-over points) from a liquid-like (G″ > G′) to a solid-like (G′ > G″) state occurred at a lower frequency (ω_x_ = 1 rad s^−1^) for H-MM than I-MM (ω_x_ = 38.4 rad s^−1^), as expected. 

The n-HA and mixed-HA structures exhibited gel-type mechanical spectra, i.e., G′ > G″ throughout the studied range, with a low degree of frequency dependence Figure 1c,d. The mechanical properties of the structures in terms of (n) values in the Ostwald-de-Waele power law (η = K·γ^n−1^), G′, G″, tan δ = G″/G′ and the B parameter for the relationship G′ = A.ω^B^, which determines the degree of frequency dependence were determined. The flow index (n < 1) indicates non-Newtonian pseudoplastic behavior. The B values define the strength and nature of the gels. It is known that B = 0 for a covalent gel, whereas B > 0 for physical gels, according to Khondkar et al. [34]. 

A strong shear thinning was observed at higher shear rates with values of flow indexes (n) as low as 0.13 Pas, indicating non-Newtonian pseudoplastic behavior for the f-HAs. The contributions of the modules were similar for the crosslinked nanoparticles, while the viscous module contribution was much lower than the elastic module for the mixed-HA system. Although similar tan δ = G″/G′~0.3, the order of magnitude of the individual values for mixed-HA was 10 times lower than that of the crosslinked nanoparticles and I-MM at the plateau. The gels were covalent type. 

Table 2 shows the viscosities of HA formulations as a function of the concentration. 

### 3.3. Mucoadhesion Functionality in vitro

**Figure 2:**
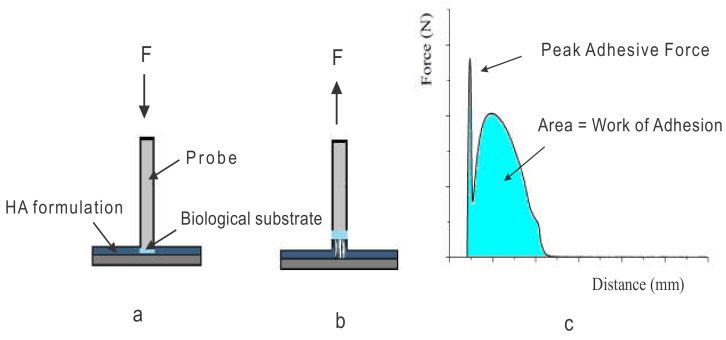
Scheme of a typical tack adhesion system during (**a**) contact and (**b**) separation steps. (**c**) A typical force vs. distance curve obtained from a tack measurement.

The mucoadhesion measurements were accomplished as described in Section 2.5. Figure 2 shows the mucoadhesion assay, the profiles of force vs. distance, and the determined parameters of the peak force, the maximum force of detachment (F_adh_), and the work of adhesion (W_adh_) or adhesion energy as the integral of the resulting force–distance profile. The measured adhesion depends on the probe speed, contact time, and force, as well as the probe shape and surface characteristics [33]. Here, assays were carried out with a mucin II tablet as a probe or a viable rat intestinal mucosa attached to a rigid probe.

#### 3.3.1. Mucoadhesion in Mucin III Tablets

**Figure 3:**
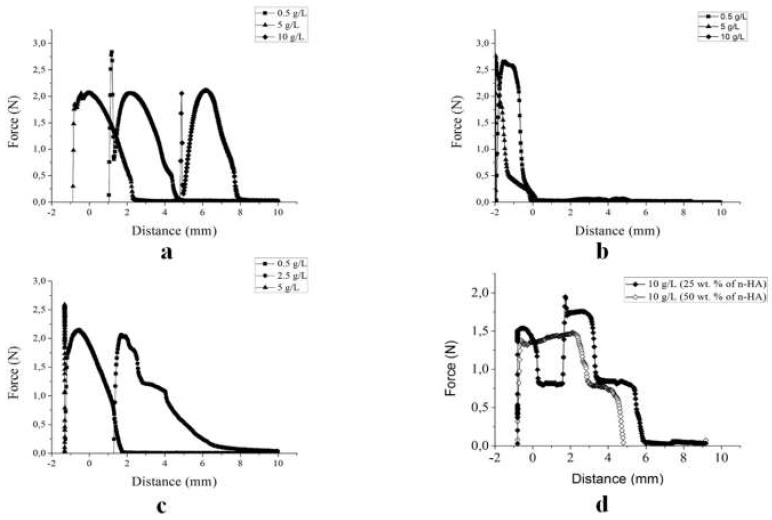
Profiles of mucoadhesion of HA dispersions on the mucin tablets. (**a**) I-MM HA (**b**) H-MM, (**c**) nanoparticles (**c**) and (**d**) mixed, at compositions of 0.5 g/L (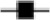), 2.5 g/L (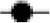), 5 g/L (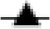) and 10 g/L (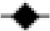,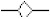 ).

Figure 3 shows the profiles obtained from the mucoadhesion assays. For I-MM at 10 g/L and 5 g/L composition, the W_adh_ was achieved by removing the probe from the viscous medium (Figure 3a). However, in Figure 3b, for H-MM, the events occurred outside the formulation (negative distance values) in the compositional range studied. Nanoparticles at 2.5 g/L performed a W_adh_ at 8 mm depth, meaning that this composition reached an enhanced depth in the mucin network. F_adh_ and W_adh_ occurred outside the penetration depth at 0.5 g/L and 5 g/L with the formation of overlapping peaks (Figure 3c). For the mixed formulation (Figure 3d) this occurred at a single concentration (10 g/L) and two compositions of nanoparticles 25% and 50% (wt.) exhibited two stages of detachment. In the first stage, from 6 mm to 2 mm of depth, whereas in the second stage it occurred at over 2 mm up to negative distance values (i.e., above the surface). 

Table S1 shows the F_adh_ (N) and W_adh_ (N.mm) as a function of the concentration of HA in the dispersions. An intra- and inter-assay statistical analysis was conducted using ANOVA with post-hoc Tukey’s multiple comparisons test. Most of the results were not significantly different. Intra-assay significant statistical difference was identified for F_adh_ at a more diluted composition (0.5 g/L) for the I-MM formulation. There was a statistically significant difference in the inter-assay for W_adh_ in mixed-HA at 25 wt.%.

#### 3.3.2. Mucoadhesion in Rat Intestinal Mucosa

**Figure 4:**
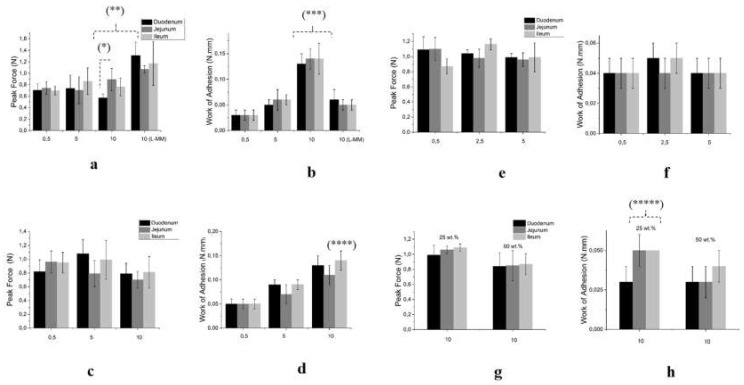
Mean values of the mucoadhesion parameters of the structures in the intestinal segments. (**a**,**b**) I-MM peak force and work of adhesion, (**c**,**d**) H-MM peak force and work of adhesion (**e**,**f**) n-HA peak force and work of adhesion, and (**g**,**h**) mixed-HA peak force and work of adhesion. Values are summarized as mean ± S.D. (*) refers to a difference between means between the duodenum and jejunum. (**) refers to inter-assay difference of means from L-MM at 10 g/L and other compositions. (***) refers to inter-assay difference of means from I-MM at 10 g/L and other compositions. (****) refers to intra-assay difference of means from H-MM at 10 g/L in ileum segment and other compositions. (*****) refers to inter-assay difference of means from mixed-HA (25 wt.% n-HA) at 10 g/L and other compositions.

**Figure 5:**
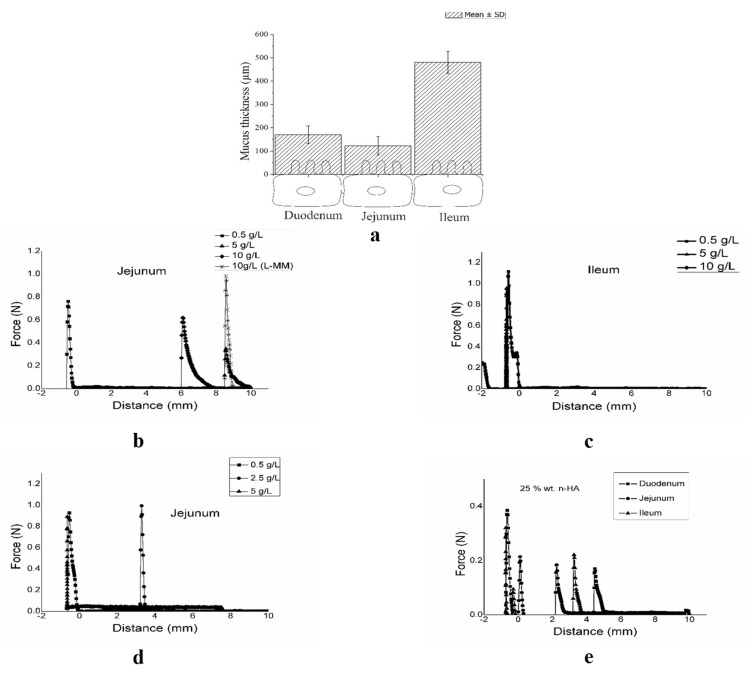
The top image shows the variation in the thickness of the mucus layer in the three different intestinal segments: Duodenum, jejunum, and ileum (**a**). Mucoadhesion profiles of intestinal segments that present the peak force in the presence of f-HA I-MM (**b**), f-HA H-MM (**c**), f-HA n-HA (**d**), and mixed-HA (**e**). Variations of the force for the detachment of the HA structures adhered to rat intestinal mucosa. The force was a function of upward probe distance for the following compositions: 0.5 g/L (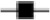), 2.5 g/L (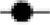), 5 g/L (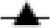), 10 g/L (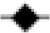), 10 g/L hydrolyzed (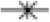), and 25 wt.% n-HA 10 g/L.

Figure 4 shows the values of the peak force F_adh_ and adhesion work W_adh_ in intestinal segments for the HA dispersions in the range of concentrations studied. Figure 5 shows the profiles of force vs. distance in the intestinal segments where the parameters presented significant differences. 

Figure 4a,b shows the highest values for F_adh_ and W_adh_ in the jejunum for I-MM HA. L-MM presented a higher peak force, but the lowest W_adh_. Figure 5a shows the force vs. distance profiles at the studied concentrations, evidencing that the adhesion curves were inside the dispersions at 10g/L in the jejunum for both dispersions. 

F_adh_ for free H-MM was not responsive at the compositional range studied, while the W_adh_ was significantly different at 10 g/L in ileum only (Figure 4c,d). However, the profiles were above the surface (Figure 5b). Figure 4e,f summarizes the profiles of F_adh_ and W_adh_ for the HA nanoparticles. No statistical difference intra- and inter-assay was identified. The profiles in jejunum for 0.5 g/L and 5 g/L overlapped outside the surface (Figure 5c). Figure 4g,h shows a significant difference for W_adh_ in the jejunum and ileum for mixed-HA dispersion containing 25 wt.% nanoparticles at 10 g/L. Figure 5d shows the profiles for 25 wt.% nanoparticles at 10 g/L. The profiles indicate that mixed-HA interpenetrate the three intestinal segments, and penetrate jejunum and ileum the most.

### 3.4. Intestinal Perfusion and Improved In Situ Single-Pass Intestinal Permeability

**Figure 6:**
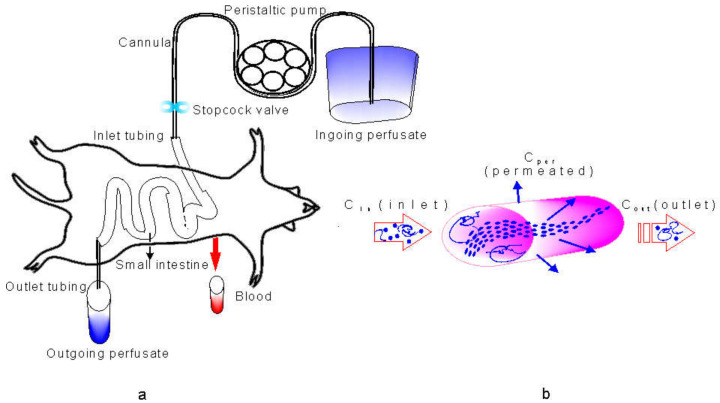
Scheme illustrative of the improved in situ single-pass intestinal perfusion (SPIP) method that uses the whole length of the animal intestine (**a**), and the fluxes involved in continuous intestinal perfusion (**b**).

The improved single-pass intestinal perfusion (SPIP) method, which considers the whole length of the animal intestine, was used to assesses the perfusates and effective intestinal permeability (P_eff_) of the structures in vivo. Figure 6 illustrates fluxes involved in the continuous intestinal perfusion and the improved in situ SPIP. Initially, HA is tangentially fed across the intestinal lumen. The microvilli retain the structures that permeate the membrane by paracellular transport, which is regulated by tight junctions, while the carrier-mediated transcellular transport is activated by the receptors and the interacting structures [35].

#### Physicochemical Characterization of Perfusate HAs

Table 3 shows the molar mass (MM) distribution of f-HA in the classes of 10^6^, 10^5^, and 10^4^ Da. There were changes in the ingoing and outgoing perfusate as a consequence of the permeation of the MM classes. The values indicate that 10^5^ Da was the best class of permeated MM (40% ± 4%), followed by 10^4^ Da (20% ± 1%) and 10^6^ Da (6% ± 0%). Furthermore, the permeability coefficients (P_eff_) that characterize the HA intestinal uptake for the studied structures in the small intestine wall of rats were calculated.

The free I-MM class from 10^5^ Da presented a higher percentage of permeation in relation to the other MM classes. However, the heterogeneity of MM distribution reduced the effective permeability of f-HA. On the other hand, the homogeneity and reduced sizes of n-HA positively impacted its permeability, raising P_eff_. Mixed-HA showed a slight improvement in the percentage permeated; however, its P_eff_ did not differ from f-HA.

**Figure 7:**
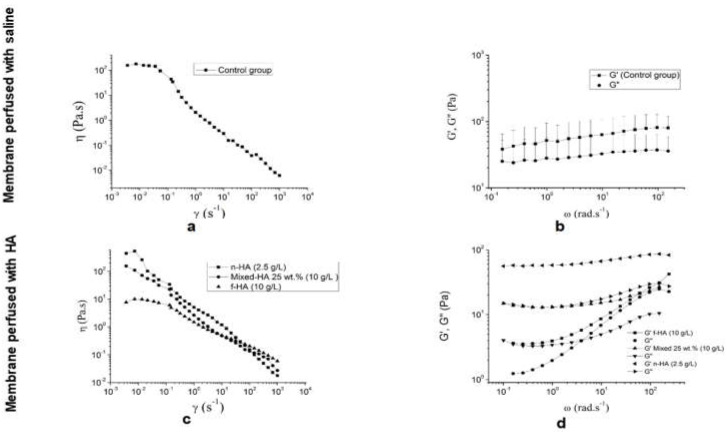
Profiles of shear viscosity (**a**,**c**) and viscous and viscoelastic modulus (**b**,**d**) of macerated small intestine of rats emptied after perfusion with saline solution (control) (**a**,**b**) and the HA formulations (**c**,**d**). Error bars represent SD = 3.

Figure 7a–d shows the shear viscosity profiles as well as the viscous and viscoelastic modulus of the macerated small intestine of rats before and after perfusion. The control group corresponds to the intestine that was perfused with a normal saline solution.

Figure 7a shows the pseudoplastic behavior and shear-thinning (shear viscosity decreasing with shear rate) of the intestinal membrane macerate before perfusion with the structures. This macerate also has an elastic gel behavior, with G′ > G″ and constant in the frequency range studied (Figure 7b). The profiles of the membrane macerate rat small intestine perfused with the HA formulations are shown in Figure 7c,d.

**Figure 8:**
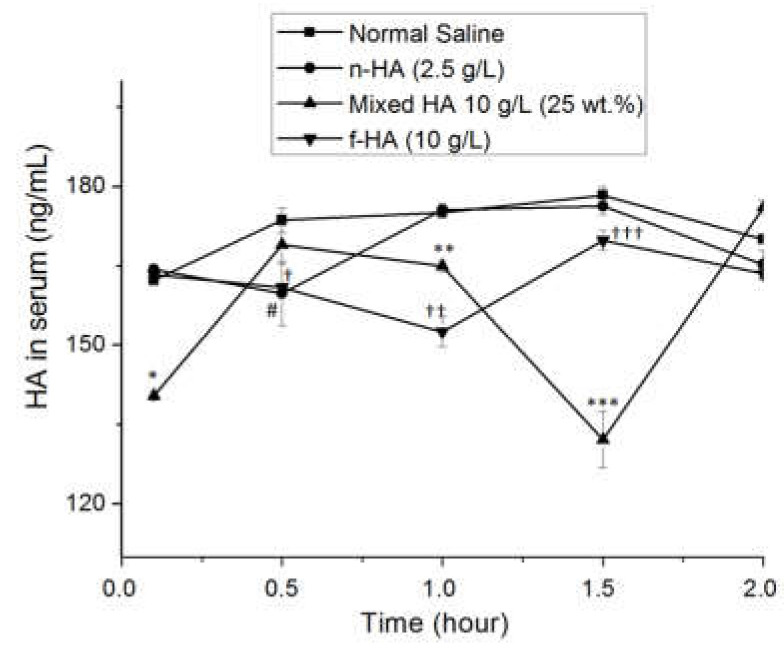
Kinetic behavior of HA clearance in the serum of rats perfused with HA formulations. The cumulative exogenous HA in the serum pool during perfusion. Error bars represent (standard deviation) SD = 3. The Student’s *t*-test was used to analyze data for significant differences. Values of *p* < 0.05 were regarded as significant. Significant statistical difference was identified for HA formulations: ^†,†† and †††^ *p* < 0.05 for f-HA (10 g/L), ^#^ *p* < 0.05 for n-HA (2.5 g/L), *, ** and *** *p* < 0.05 for Mixed HA 25 wt.% n-HA (10 g/L), compared to the same time point in the normal saline control.

Figure 8 shows the clearance kinetics for the exogenous HA absorbed. The exogenous HA is removed from the bloodstream, never exceeding endogenous levels (standard saline group control). The clearance of n-HA occurred in little time (0.5 h), while the plasmatic clearance of I-MM HA structures occurred within 1 h, and the clearance of mixed-HA started earlier (before 6 min), ending within 1.5 h. The concentration of HA in the blood tended to normalize after the period of clearance of exogenous HA, recovering homeostasis within 2 h.

## 4. Discussion

In previous work, we reviewed the structural changes of HA related to concentration, MM, pH, the gastrointestinal system and the molecular dynamics of intestinal uptake and signaling, immunomodulation at intestinal and systemic levels, and HA fate in other tissues. Detailed studies still need to be devised to determine the effect of the physicochemical properties of HA in the formation of TLR4/CD44 clusters in the intestinal environment. The nanotechnological approach represents progress in the field of oral HA administration [36]. Here, we carried out an experimental investigation on the physicochemical, rheological, and in vitro mucoadhesion properties of the HA structures and their influence on the intestinal uptake in vivo. Dispersions containing high (H-MM HA), intermediate (I-MM HA), and low (L-MM HA) molar mass HA (f-HA) were prepared and characterized, as well as dispersions of ADH crosslinked nanoparticles (n-HA) and containing mixed structures composed of I-MM HA + n-HA (mixed-HA). The physicochemical characterization showed that f-HA chains form colloidal structures with physical entanglement. The hydrodynamic diameter, size distribution, and zeta potential of the structures were controlled by concentration, MM, density, viscosity, and dissociation of the carboxyl groups on the surface. MM distribution was crucial to the architecture of the structures. Table 1 shows the results in a semi-diluted state (0.2 g/L) for the studied structures. H-MM HA formed larger and more stable structures (lower Z-average and PDI) due to a greater amount of physical crosslinking [31,37,38] and larger chains of the 10^6^ Da fraction (Table 1). The hydrodynamic diameter distribution showed sizes predominantly in the 200 nm class (N-distribution) but also in the 100 nm class (I-distribution) (Figure S1a,b, Supplementary Information). The denser core inside the structure of H-MM HA provided a higher exposition of the carboxyl groups on the surface of the structures. However, the high density of the groups and the viscosity of the dispersions made its dissociation difficult, providing the lowest zeta potential (ZP) (−4.9 ± 1.7 mV). ZP determines the electrostatic potential at the slipping plane, which includes the Debye length, that represents the length of the double electric layer provided by the ions around a particle. ZP represents the charge exposed to the medium that determines the adhesion behavior of the HA structures on the surfaces in vitro and in vivo [34,39]. Therefore, ZP reflects the differences among materials, such as structure, architecture and surface charge due to different MM HA. An abrupt increase in the absolute value of ZP (−40 ± 6.1 mV) was observed for I-MM HA.The reduction of the physical crosslinks distributed the carboxyl groups inside and on the surface, and the lower viscosity promoted its dissociation, which explains the determined ZP (Table 1). Furthermore, I-MM HA formed less dense, more hydrated, and flexible structures, explained by the high SD in Z-average 538.3 ± 183.5 and the size distribution in the I-distribution (Figure S1c, Supplementary Information). As the scattering of light is proportional to the density of the structure [24], the predominant size was 10 nm according to (N) distributions (Figure S1d, Supplementary Information). L-MM had a smaller average MM and a structure similar to I-MM, with the carboxyl groups at the surface (ZP= −24 ± 5.7), but with a lower Z-average (345.7 ± 105.3) and distribution due to the shorter chains. A predominant peak was observed at 50 nm (Figure S1e,f, Supplementary Information).

HA crosslinked nanoparticles at semi-dilute regimes, 0.2 g/L, and at 0.5 g/L, presented sizes dependent on concentrations. The crosslinking reduced the size dispersion compared to free I-MM, as expected. Furthermore, the crosslinking with ADH via EDC (reaction with carboxyl groups) also reduced the negative charge on the surface yielding lower ZP compared to I-MM (from −40.0 ± 6.1 to −20.0 ± 5.7 mV). Although the stability criterion, ZP > 30 mV or ZP < −30 mV [39], the reduction in ZP does not severely compromise the electrostatic stability of the nanoparticles.

Free I-MM was mixed with 25 or 50 wt.% of nanoparticles to obtain mixed HA structures. Both compositions were prepared in concentrated form (10 g/L). At the lower concentration (25 wt.%), the interactions between free HA and the nanoparticles formed large, dense, unstable, and heterogeneous structures with a Z-average of 928.8 ± 170.9, a predominant size of 342 nm (Figure S2g,h), and ZP of −7.41 mV. However, when the nanoparticle concentration was increased to 50 wt.%, the Z-average changed to 2652 ± 222.1 nm with a predominant size of 10 nm and ZP of −11.60 ± 0.70 mV. From these results, it can be inferred the mixed structure contained heterogeneous and highly hydrated domains filled with nanoparticles.

The rheological characterization showed a pseudoplastic microstructural behavior with a lower “n” index and lower crossover point for H-MM than I-MM, indicating that H-MM HA can be used at lower concentrations to obtain the same rheological effects (Figure 1a,b). As a consequence, H-MM HA has a higher relaxation time due to the higher fraction of 10^6^ Da. The nanoparticles produced a weak gel-like structure with viscous (G″) and elastic components (G′), and tan δ = G″/G′~0.3. Although they had a similar value for tan δ, both moduli were reduced for the mixed-HA structures (Figure 1c,d). Table 2 shows that the apparent viscosity decreased with MM of the structures, as expected. The structuration in crosslinked nanoparticles produced less viscous dispersions. Similar values were observed to the apparent viscosity of the mixed-HA structures, justifying the smaller and lesser flexible domains compared to free I-MM, due to the presence of the nanoparticles.

The capability for adhesion—the first step for intestinal uptake—was initially assayed in vitro in mucin. It is noted that the native mucus concentration was greater than 20 mg/mL [40]. Thus, it is clear that mucins in the native state are well entangled. Mucoadhesion is a function of various factors, such as electrostatic charge, hydration, hydrophobic interactions, and the rheological behavior of the dispersions, so that the structures play an important role. In particular, the abundance of COOH groups promote adhesion through hydrogen bond formation with biological substrates [41].

One common criterion for material with good adhesive properties is an elastic modulus of less than 10^5^ Pa [42]. Materials with elastic moduli exceeding the Dahlquist criterion have poor adhesive characteristics due to their inability to dissipate energy via viscous contributions or to deform to make good contact with a surface [43]. In biological materials, the magnitude of the elastic modulus was much lower (G′ = 10 for n-HA and G′ = 1 for mixed-HA), as expected.

The mucoadhesion assays carried out using a mucin tablets probe showed that all systems were responsive to detachment force (F_adh_) and adhesion work (W_adh_) (Table S1), with similar values for the assayed concentrations. However, the force vs. distance profiles showed different mucoadhesion mechanisms (Figure 3). The profiles of H-MM had F_adh_ and W_adh_ out of the probe path (negative values), inferring the presence of fibrils at the surface before detachment [44]. Therefore, the cohesion of HA chains in the larger and denser structures of H-MM did not penetrate the mucin network (Figure 3a). The profiles of I-MM HA (10 g/L) showed that the hydration capacity and flexibility of the structures provided penetration of HA into the mucin network, counterbalancing electrostatic repulsion (Figure 3b). The nanoparticles interacted with mucin into dispersion at 2.5 g/L only. The profiles of n-HA and mixed-HA showed detachment in steps, indicating different interaction mechanisms compared to I-MM HA. It can be inferred that the size and stability produced by the crosslinking allowed the nanoparticles to penetrate deeper into the mucin network (20–200 nm cut-off pores). Furthermore, the gel’s behavior with the elastic and viscous components promoted the retention and interaction of energy with n-HA and mixed-HA structures.

With the rat intestinal membrane covering the probe, there was a difference in the responses among the duodenum, jejunum, and ileum due to their variable thicknesses (Figure 4 and Figure 5). The tendency of the responses in the scope of the assays was similar to those obtained by the mucin probe, with significant differences for I-MM HA (10 g/L), n-HA (2.5 g/L), and mixed-HA (25 wt.%). The effects were pronounced in the jejunum segment for I-MM and n-HA, and along the small intestine for mixed-HA. When using the intestinal membrane, the cellular viability [45] and the functionality of the TLR4 receptors from the freshly excised mucosa raise new questions. Stabilization of the binding of HA structures provides synergistic effects on TLR4 receptors [46]. The HA structures (roughly 65 nm) in the specific MM range (100–300 kDa) positively influenced the structuring of multiple HA receptors into the transmembrane assembly, essential to enable the stabilization of binding [47].

By measuring HA concentrations in ingoing and outgoing fluxes, the effective permeability coefficients (P_eff_) that characterize HA intestinal uptake of the structures in the small intestine wall of rats were calculated. The best uptake, P_eff_ 2.62 × 10^−5^ mm/min, which corresponds to 80% ± 5% permeated mass, was obtained with the n-HA structure, while the f-HA and mixed-HA structures were more poorly permeated (Table 3).

The rheology of the intestinal membrane after perfusions showed no significant differences in pseudoplasticity for the intestinal membrane perfused with n-HA or mixed-HA, but changes could be observed for IMM-HA (Figure 7b) compared to the membrane perfused with saline as a control (Figure 7a). Oscillatory measurements in the membrane macerate showed that the membrane retained its viscoelastic behavior after perfusion with the n-HA structure. However, the gel-like behavior was lost after IMM-HA perfusion and was much weaker for mixed-HA structure. These results explain the significant difference in the effective permeability coefficients for n-HA compared to the other structures. Moreover, these results confirmed the behavior inferred from the in vitro assays. Therefore, most of the IMM-HA and mixed-HA structures diffused slowly due to the tight junctions sealing the enterocytes in the membrane. Hisada et al. [44] found that the transmural flux of free HA (5 mg/mL) and MM < 5 kDa in a monolayer of Caco-2 cells was 0.04% (m/v), and it was size-dependent.

The paracellular transport of free macromolecules larger than 250–300 kDa makes only a minor contribution to overall intestinal permeation [48,49]. However, it has been suggested that the structuring in nanoparticles is largely absorbed [50]. Nanoparticles can be substrates to TLR4 intestinal transporter protein in a carrier-mediated uptake of HA larger than 250–300 kDa and with a specific diameter size of roughly 65 nm [35,47,51]. Therefore, the n-HA structure may be diffused by paracellular and transcellular mechanisms, thus promoting its uptake into the intestinal mucosa.

After uptake, the TLR4 receptor releases the intact HA to the Peyer’s patches, following from the lymphoid follicles to the systemic bloodstream [52]. HA concentrations in serum were also analyzed. As known, the levels of exogenous HA in the bloodstream cannot exceed endogenous levels, due to the deleterious effect on plasma even for low MM. Studies have shown that the removal of exogenous HA from the bloodstream compartment occurs from 2 to 6 min after intravenous administration in the healthy adult human, and residual fractions were degraded in the lymph nodes and excreted by the kidneys at a three-fold clearance to the urinary tract in relation to total systemic turnover [53].

Figure 8 shows that exogenous absorbed HA is rapidly removed from the bloodstream, never exceeding endogenous levels (standard saline group control). By analyzing the clearance time, the profiles showed the clearance of n-HA occurred in a short time (0.5 h). Barua and Mitragotri [54] reported that stable and smaller sizes (<100 nm) from crosslinked HA permeated the vascular endothelium in less time due to the higher rates of endocytosis and more rapid lymphatic transport. Plasmatic clearance of the IMM-HA structure occurred within 1 h, due to its slow intestinal uptake, while the clearance of mixed-HA began earlier (before 6 min), probably due to the smaller compositional fraction (25 wt.%) of nanoparticles, ending within 1.5 h, due to the presence of f-HA. The concentration of HA in the blood tended to normalize after the period of clearance of exogenous HA, recovering homeostasis within 2 h.

In summary, the in vivo data highlight the promise of HA structures for specific functions in the intestinal tract. As I-MM HA (mainly 10^5^ Da) is preferably absorbed (Table 1) and remains attached to the mucus nanostructure (Figure 7b), it is prone to pre-systemic metabolism (cleavage/disintegration) due to variations in pH and hydrolysis caused by microbiota enzymes, making it a promising formulation for dysbiosis treatment. In terms of reaching other tissues, the n-HA structures are more effective because they have the highest permeability coefficient in the intestinal membrane and have a shorter clearance time in the bloodstream (Figure 8).

## 5. Conclusions

HA structure, rheology, and mucoadhesion have play a key influence on interactions and uptake in the intestinal membrane. The MM distribution determines the architecture of the structures, with consequences on the surface and rheological properties that modulate mucoadhesion. H-MM was not responsive to mucoadhesion forces in the intestinal segments, while the adhesion work was significantly different at 10 g/L in ileum only. At the same concentration, I-MM and L-MM adhered to rat intestinal mucosa in the jejunum. HA nanoparticles had no significant adhesion while mixed-HA interpenetrate the three intestinal segments, and penetrate jejunum and ileum mainly. The tendencies observed from in vitro assays were confirmed using in vivo assays. The plasmatic clearance of n-HA occurred in the shortest time, followed by I-MM HA and mixed HA. Therefore, the free structure of I-MM HA is adequate for dysbiosis treatments, while the n-HA structure is promising to reach other tissues. These results shed light on the development of HA formulations for drug delivery via oral administration.

## Figures and Tables

**Table 1:** Average hydrodynamic size (Z-average), zeta potential (ZP), average molar mass (MM), polydispersity index (PDI) and MM distribution: Free hyaluronic acids (HAs) (f-HA) with high (H-MM), intermediate (I-MM), and low (L-MM) molar mass (MM); the crosslinked HA nanoparticles (n-HA) and the mixed formulations (nanoparticles + IMM − HA) (mixed-HA) with 25% or 50% (wt.) of nanoparticles. All formulations were buffered in pH 7.4 aqueous dispersions. (-) not determined.

Property	Free HA			n-HA	Mixed-HA
	H-MM	I-MM	L-MM		
Z-average	114.0 ± 14.51	538.3 ± 186.5	345.7 ± 105.3	268.7 ± 3.57	928.8 ± 170.9
ZP (mV)	−4.9 ± 1.7	−40.0 ± 6.1	−24.0 ± 5.7	−20.0 ± 0.14	−7.41 ± 0.0
Average MM (×10^5^), (Da)	7.2	5.2	0.08	-	-
PDI	0.59 ± 0.04	0.66 ± 0.12	0.75 ± 0.22	0.56 ± 0.11	0.73 ± 0.07
MM Distribution, (%)				-	-
10^6^;	34.0 ± 0.4	16.0 ± 0.1	≈0		
10^5^;	50.0 ± 0.7	74.0 ± 0.8	6 ± 0.0		
10^4^ (Da)	16.0 ± 0.0	10.0 ± 0.0	94.0 ± 1.9		

L-MM: Hydrolyzed I-MM; measurements were carried out in semi-diluted states (0.2 g/L). Purity: H-MM (>95%), I-MM and L-MM (~90%). The yield in n-HA was 31.2 ± 1.1%.

**Table 2:** Viscosities of HA formulations as a function of the concentration. (f-HAs) with high (H-MM) and intermediate (I-MM) molar mass, crosslinked HA nanoparticles (n-HA) and mixed formulations (nanoparticles + IMM- HA) (mixed-HA) with 25% or 50% (wt.) of nanoparticles. (-) not determined.

HA Structure		f-HA			n-HA	Mixed-HA (%)	
		I-MM	H-MM	L-MM		25 wt.	50 wt.
		Viscosity at 25 °C (mPa·s)					
**Concentration (g/L)**	0.5	13.7	49.6	-	1.1	-	-
	2.5	-	-	-	1.8	-	-
	5	37.6	86.9	-	4.9	-	-
	10	349	390	7.8	-	1.5	0.7

**Table 3:** Ingoing and outgoing concentrations, permeate and permeability coefficient (P_eff_) as function of HA structures.

HA Structure	MM (% Class)	Ingoing	Outgoing	Permeate (%)	P_eff_ (×10^−5^ mm/min)
I-MM 10 g/L	10^6^	16 ± 0.0	15 ± 0.0	6 ± 0	0.9 ± 0.1
	10^5^	74 ± 0.8	46 ± 0.0	40 ± 4	
	10^4^	10 ± 0.0	8 ± 0.0	20 ± 1	
n-HA 2.5 g/L		2.5 ± 0.1	0.5 ± 0.0	80 ± 5	2.6 ± 0.2
Mixed-HA 10 g/L		10 ± 0.0	5.5 ± 0.0	45 ± 4	1.0 ± 0.0

Permeate (%): calculated by the difference between the ingoing and outgoing MM classes for free HA structures and by the difference between the ingoing and outgoing concentration for n-HA and Mixed-HA structures. Permeability coefficients (P_eff_) calculated according to (Eq. 2): Flow rate (mL/min): 0.5, 0.2 and 0.2, for free (10 g/L), n-HA (2.5 g/L) and mixed-HA 25 wt.% n-HA (10 g/L) respectively.

## Supplementary Materials

The following are available online at https://www.mdpi.com/2218-273X/10/10/1422/s1, Figure S1: Size distribution spectra from semi-diluted free HA, Figure S2: Size distribution spectra from HA nanoparticles, Figure S3: Profiles of mucoadhesion on the jejunum and ileum segments from free I-MM and free H-MM HA respectively, Figure S4: Profiles of mucoadhesion on the jejunum segment from HA nanoparticulate, Figure S5: Profiles of mucoadhesion on the intestinal segments from mixed HA, Figure S6: Scattering for number predominant of particle sizes, Table S1: Mean values of the mucoadhesion parameters of the structures on mucin tablets.
