# Supplementary material for: Hyaluronic Acid in the Intestinal Tract: Influence of Structure, Rheology, and Mucoadhesion on the Intestinal Uptake in Rats
Source: Biomolecules. 2020 Oct 8;10(10):1422. doi: 10.3390/biom10101422 (PMC7601924; doi:10.3390/biom10101422)
Supplement: Supplementary file 1 [file biomolecules-10-01422-s001.pdf]

Supplementary Materials

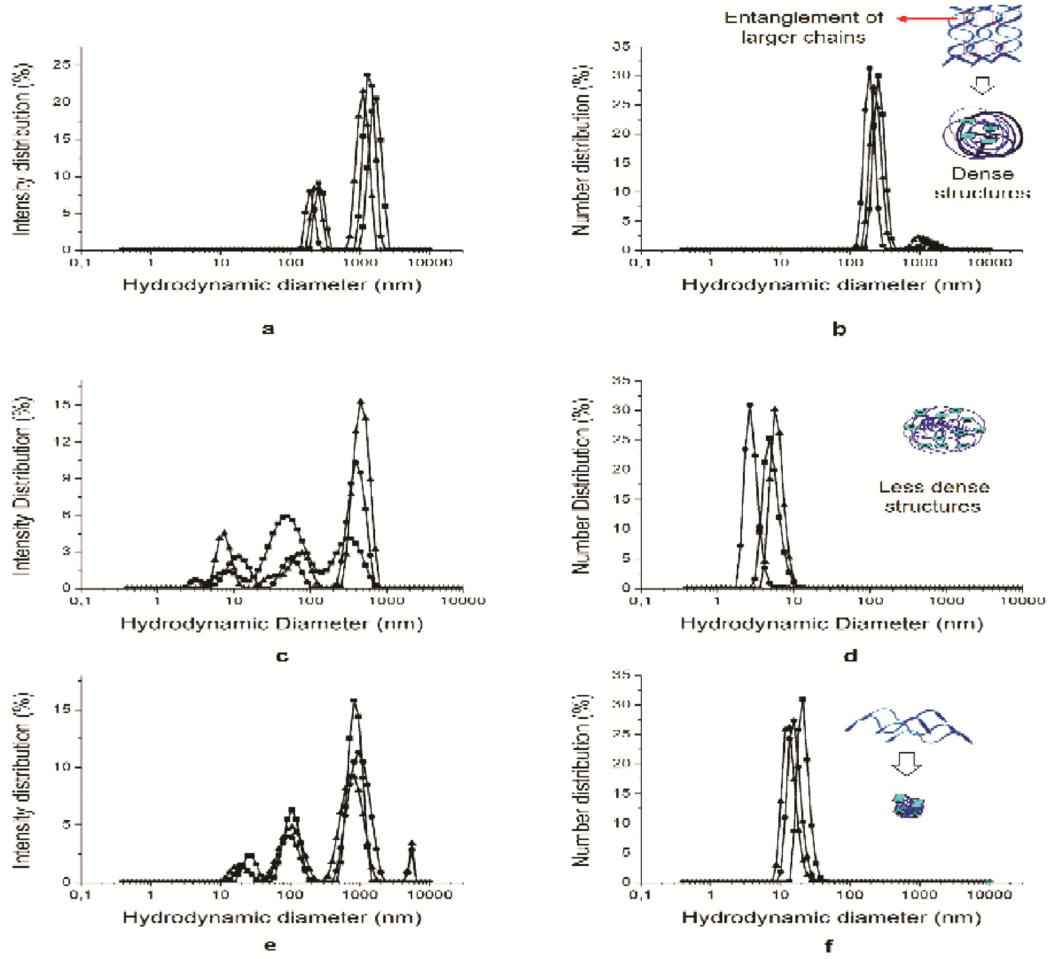

Figure S1: Size distribution spectra from semi-diluted free HA. Formulations at 0.2 g/L. (**a**,**b**) H-MM, (**c**,**d**) I-MM, and (**e**,**f**) L-MM. The samples were buffered 7.4 aqueous dispersions. The measurements were done in three independent samples. Mean diameter (Z-average); polydispersity index (PDI) were: H-MM (1386 ± 27.58; 0.89 ± 0.11); I-MM (964.2 ± 125.8; 0.80 ± 0.00) and L-MM (345.7 ± 105.3; 0.75 ± 0.22).

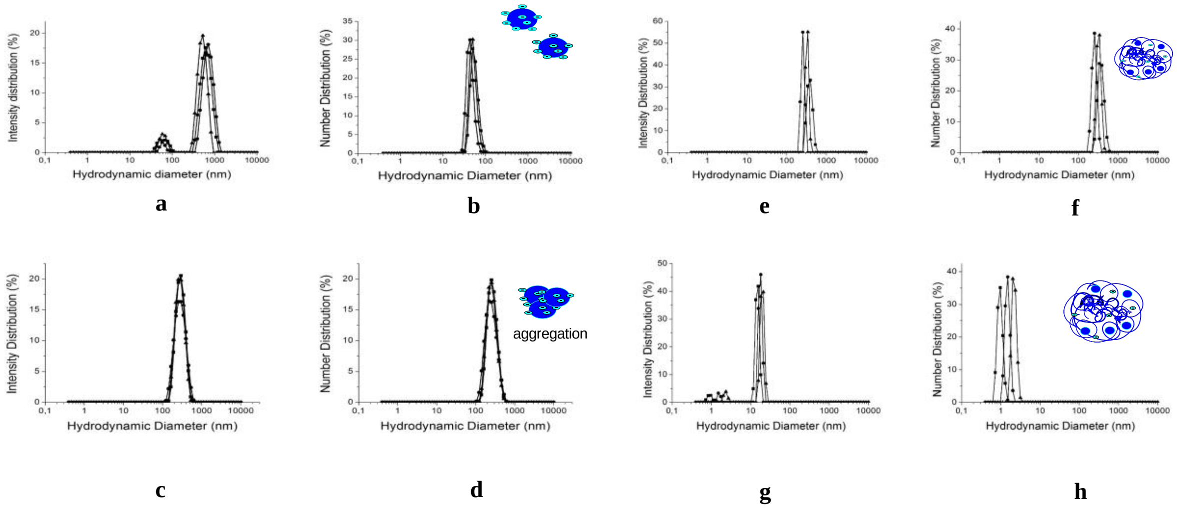

Figure S2: Size distribution spectra from HA nanoparticles. Formulations at 0.2 g/L (**a**,**b**) and 0.5 g/L (**c**,**d**). The samples were buffered 7.4 aqueous dispersions. The measurements were done in three independent samples. Mean diameter (Z-average); polydispersity index (PDI) were: (268.7 ± 3.57; 0.56 ± 0.11) for 0.2 g/L and (485.3 ± 87.61; 0.28 ± 0.04) for 0.5 g/L. Size distribution spectra from mixed HA nanostructures. Formulation of the I-MM at 10 g/L with 25 wt. % (**e**,**f**) and 50 wt. % (**g**,**h**) of nanoparticles. The samples were buffered 7.4 aqueous dispersions. The measurements were done in three independent samples. Mean diameter (Z-average); polydispersity index (PDI) were: (928.8 ± 170.9; 0.73 ± 0.07) and (2652 ± 222.1; 0.88 ± 0.13) respectively.

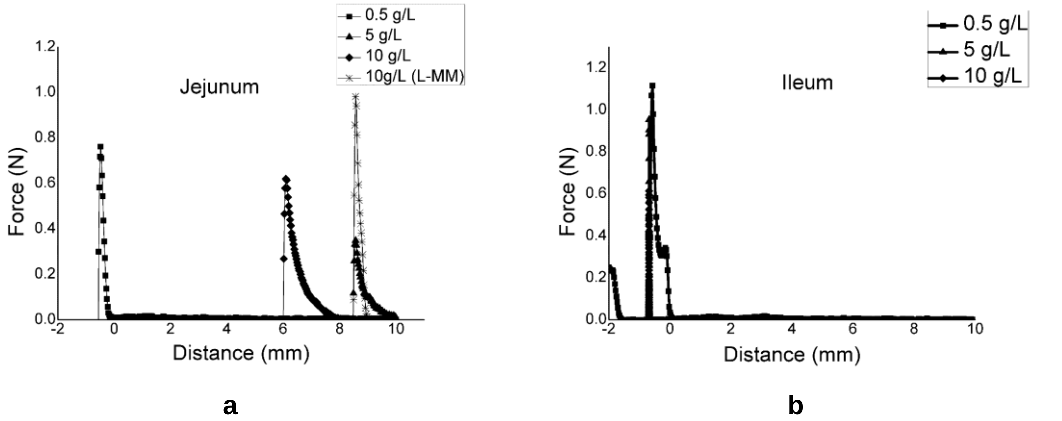

Figure S3: Profiles of mucoadhesion on the jejunum and ileum segments from free I-MM and free H-MM HA respectively. Variations of the force necessary for detachment of the free HA formulation adhered to rat intestinal mucosa. Force was a function of upward probe distance for I-MM and L-MM in (**a**) and H-MM in (**b**), to compositions: 0.5 g/L (■), 5 g/L (▲), 10 g/L (◆) and 10 g/L hydrolyzed (✳).

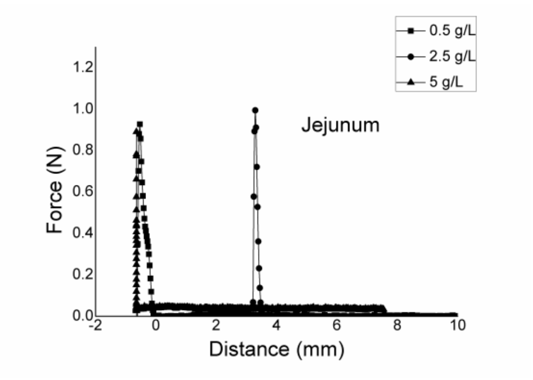

Figure S4. Profiles of mucoadhesion on the jejunum segment from HA nanoparticulate. Variations of the force for the detachment of the HA nanoparticles adhered to rat intestinal mucosa. Force was a function of upward probe distance for the compositions: 0.5 g/L (■), 2.5 g/L (●), and 5 g/L (▲).

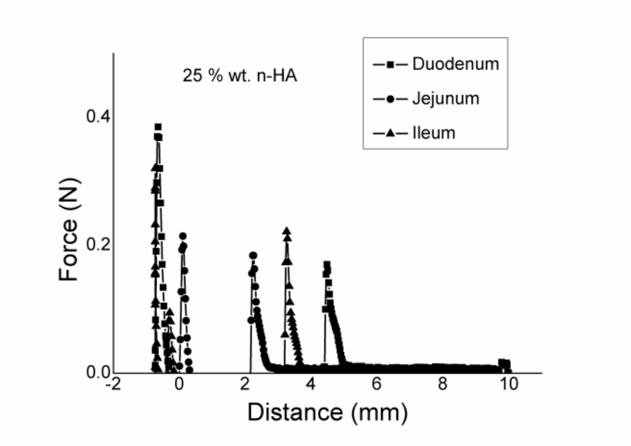

Figure S5: Profiles of mucoadhesion on the intestinal segments from mixed HA. Variations of the force necessary for detachment of the 25 wt. % mixed HA structures adhered to rat intestinal mucosa. Force was a function of upward probe distance obtained in the duodenum (-■-), jejunum (-●-) and ileum (-▲-).

- Free HA

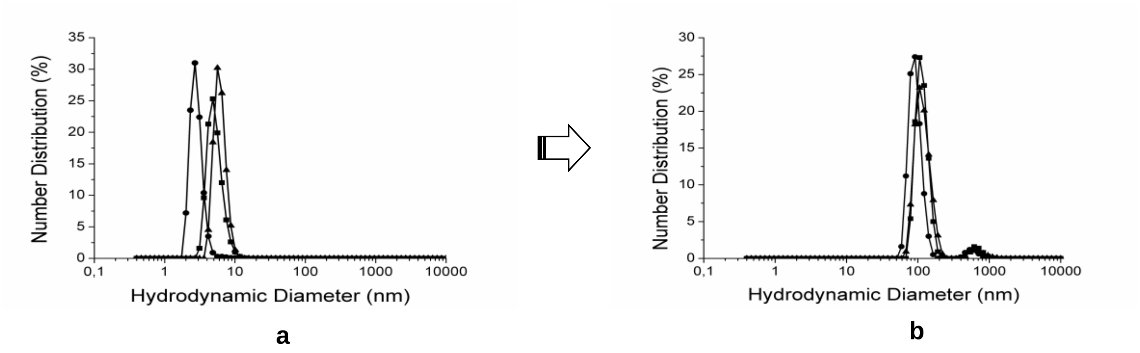

- Crosslinked HA-ADH nanoparticles

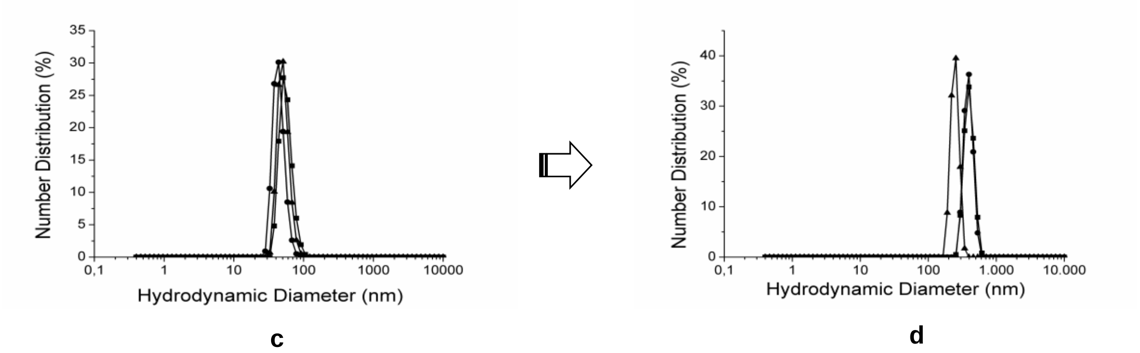

- Mix 25 wt. %

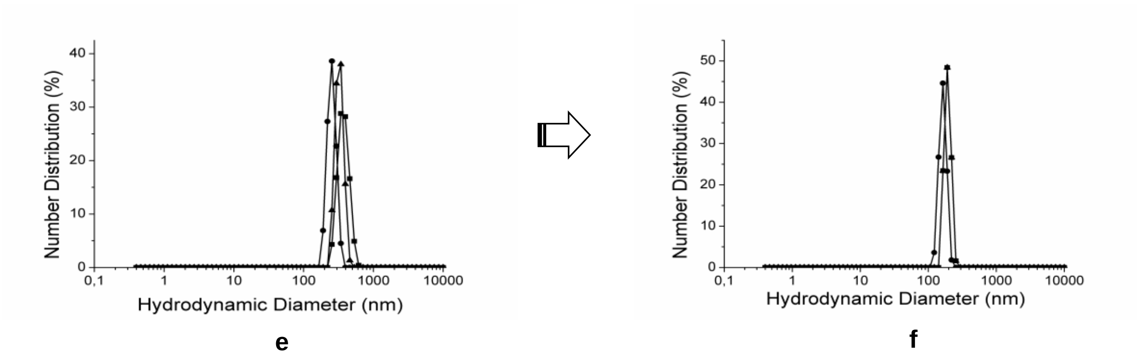

Figure S6: Scattering for number predominant of particle sizes. N- distribution spectra from the ingoing perfusate (left) and outgoing perfusate (right). The Number-distribution measurements were done in three independent samples.

Table S1: Mean values of the mucoadhesion parameters of the structures on mucin tablets. Peak force (N) and work of adhesion (N.mm) as function of concentration. The assays were carried out at the following conditions: descended constant velocity of the analytical probe 0.2 mm/s; penetration depth10 mm; downward tensile 0.49 N applied during 3 min.

**Mucin III Tablets**						
**HA Structure**		**f-HA**		**n-HA**	**Mixed -HA**	
		I-MM	H-MM		25 % (wt.)	50% (wt.)
		Fadh<br>Wadh	Fadh<br>Wadh	Fadh<br>Wadh	Fadh<br>Wadh	Fadh<br>Wadh
**Concentration (g/L)**	0.5	2.80 ± 0.06<br>0.97 ± 0.08	2.38 ± 0.28<br>0.74 ± 0.04	2.42 ± 0.27<br>0.88 ± 0.09	-	-
	2.5	-	-	2.00 ± 0.14<br>0.93 ± 0.14	-	-
	5	2.20 ±0.21<br>1.00 ± 0.05	*2.85 ± 0.10<br>*1.57 ± 0.42	2.47 ± 0.12<br>0.74 ± 0.02	-	-
	10	2.06 ± 0.06<br>0.82 ± 0.0	*2.66 ± 0.06<br>*0.86 ± 0.05	-	#2.59 ± 0.17<br>#2.55 ± 0.83	#2.77 ± 0.16<br>#2.53 ± 0.76

- Not applicable; Measurements presented as mean ± (S.D.) (*n* = 3). *Values obtained outside the penetration depth (Figures S3a,b). # Values obtained with part of the curve outside the penetration depth (Figure S5).
